# Supplementary material for: NFATc1 controls the cytotoxicity of CD8+ T cells
Source: Nat Commun. 2017 Sep 11;8:511. doi: 10.1038/s41467-017-00612-6 (PMC5593830; doi:10.1038/s41467-017-00612-6)
Supplement: Supplementary file 2 — Supplementary Information [file 41467_2017_612_MOESM2_ESM.pdf]

Description of Supplementary Files

File name: Supplementary Information
Description: Supplementary figures, supplementary table 1 and supplementary references.

File name: Peer Review File

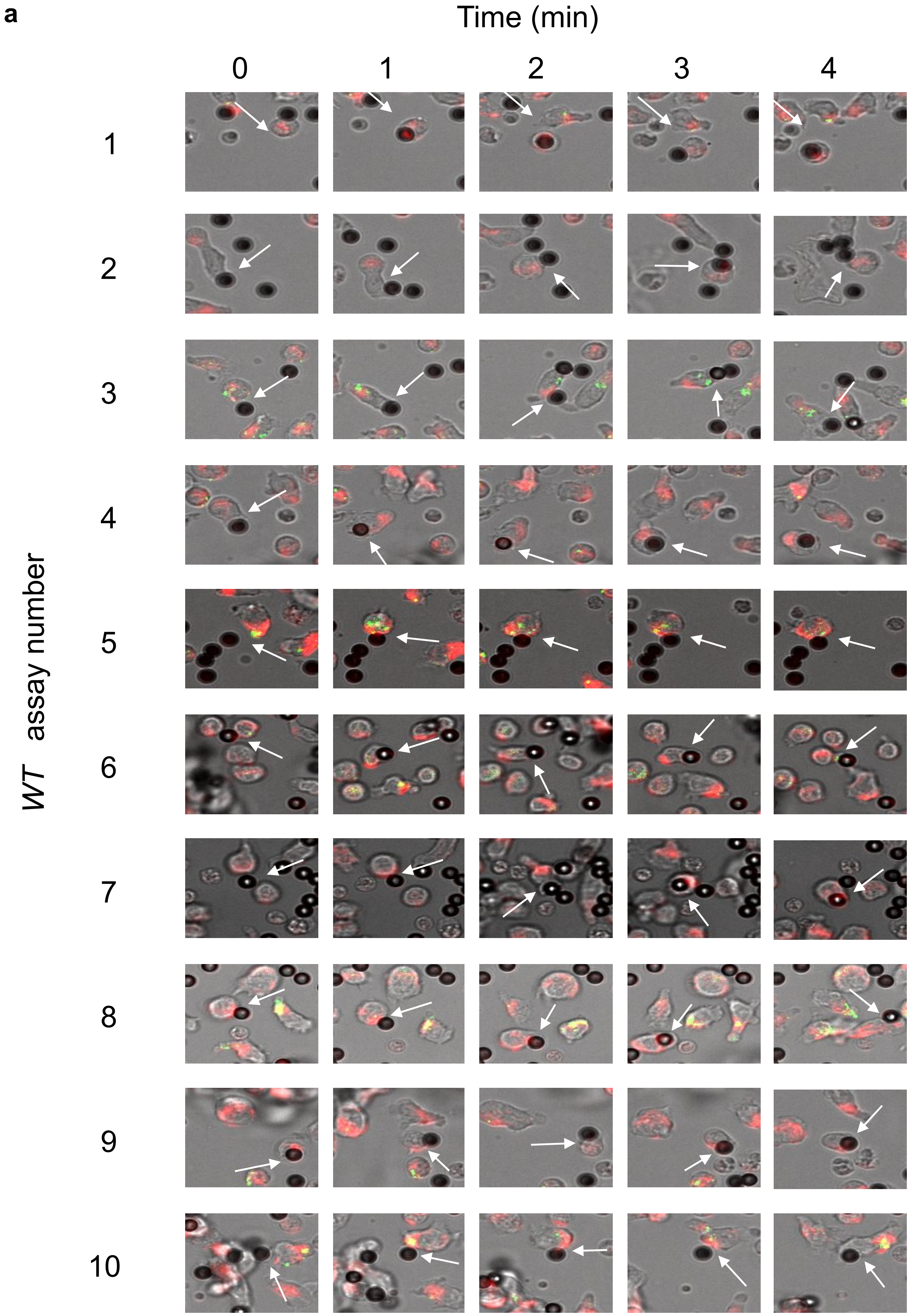

Supplementary Figure 1a

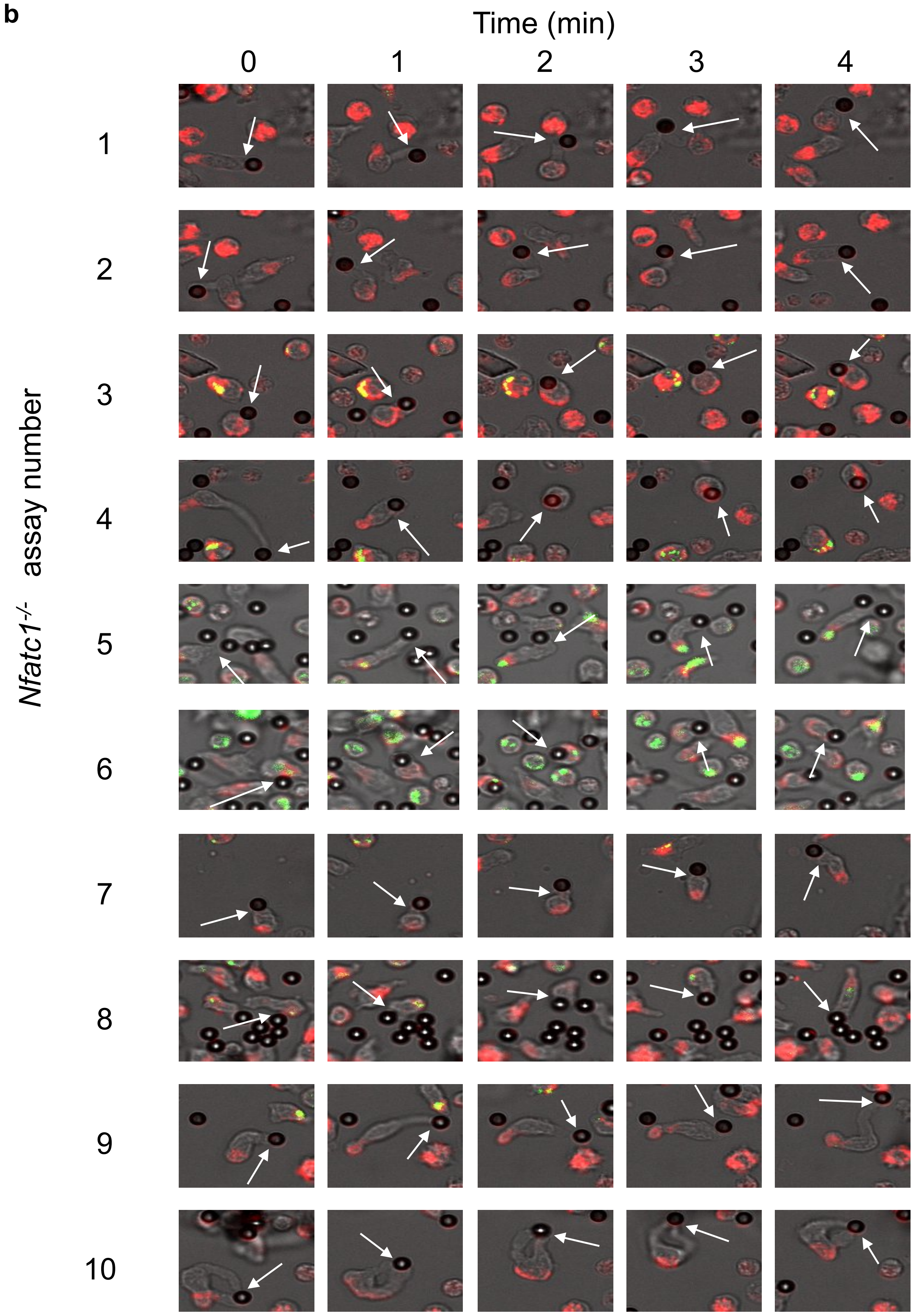

Supplementary Figure 1b

c

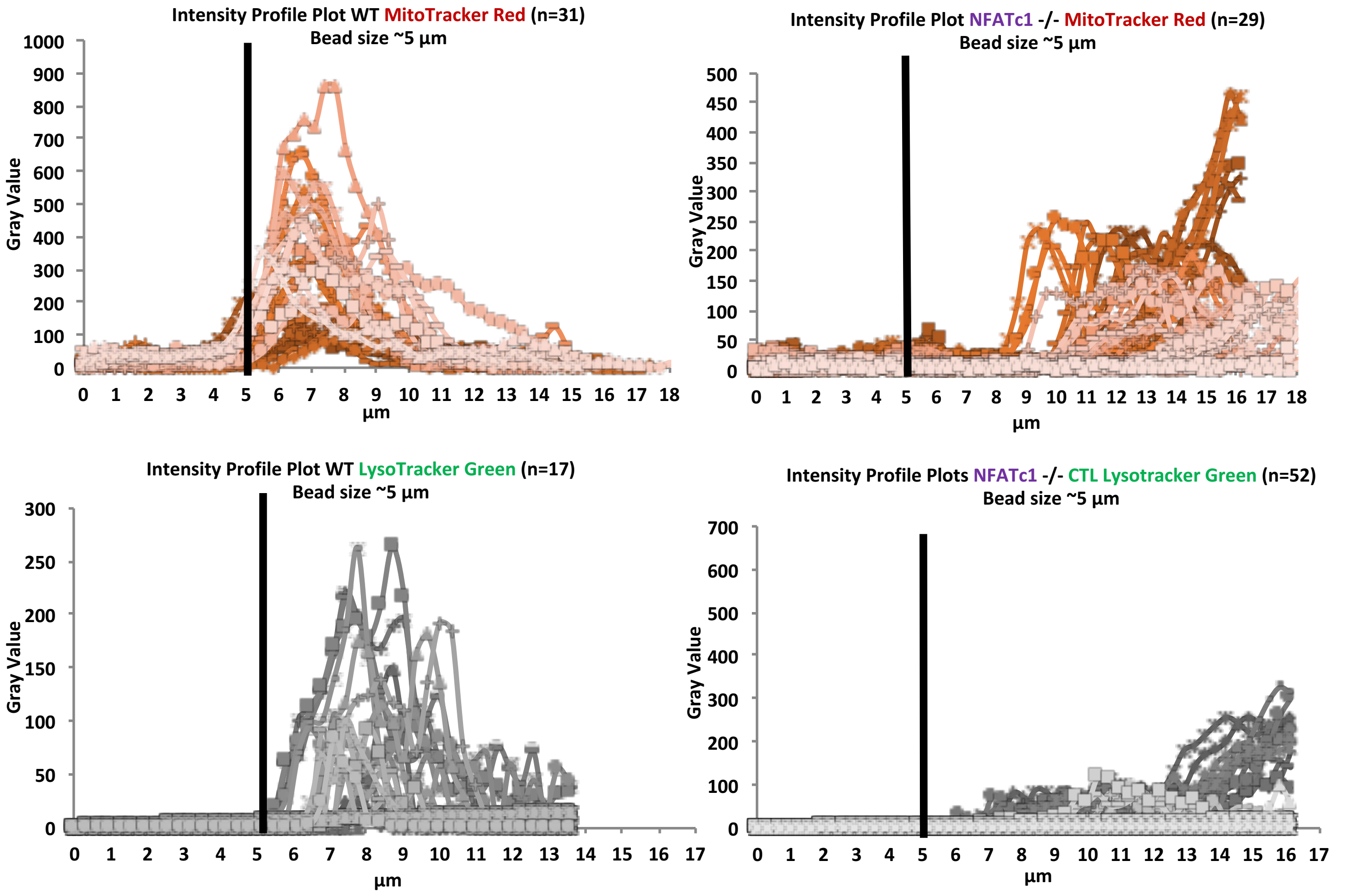

Supplementary Fig. 1 Defective polarization of lytic granules and mitochondria in NFATc1-deficient CTLs. Bright field and fluorescence microscopy of living cells showing the recruitment of lytic granules (green) and mitochondria (red) in WT (**a**) and *Nfatc1*$^{-/-}$ (**b**) CTLs upon contact with αCD3/CD28-loaded beads. Arrows indicate the contacts between beads and CTLs. (**c**) Intensity profile plots for lytic granules (LysoTracker® Green) and mitochondria (MitoTracker® Deep Red) from WT and *Nfatc1*$^{-/-}$ CTL contacting beads loaded with αCD3/CD28. Left: Distribution of organelles in WT CTLs. Right: Distribution of organelles in *Nfatc1*$^{-/-}$ CTL (3 to 5 profiles per time point, ~10 cells each condition). Black bars indicate bead-cell border (bead size 5 µm).

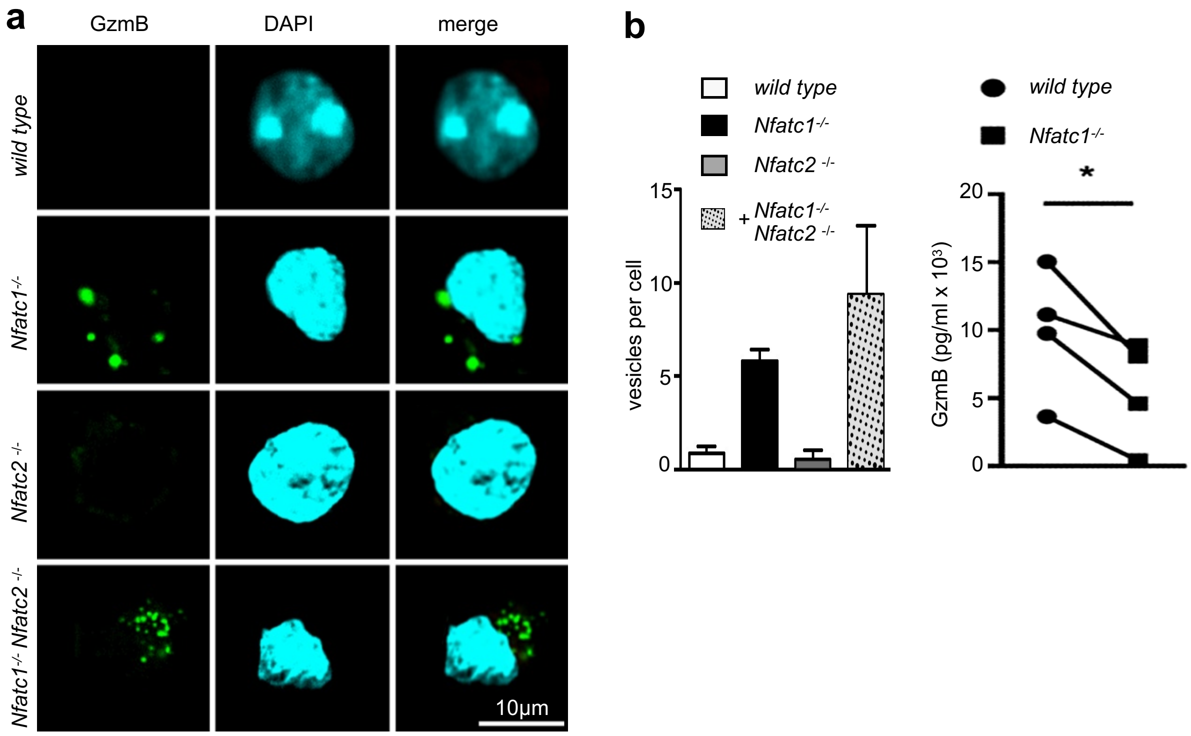

Supplementary Fig. 2 Effect of NFATc1 ablation on the accumulation of granzyme B in CTLs. (**a**) Accumulation of granzyme B-containing cytoplasmic granules in *Nfatc1*$^{-/-}$ CTLs. Confocal microscopy. **(b)** Left, column presentation showing results from two independent assays. Right, granzyme B secretion, measured by ELISAs from the supernatant of CTLs. Typical assays of 3 experiments are shown as means ± SEM.

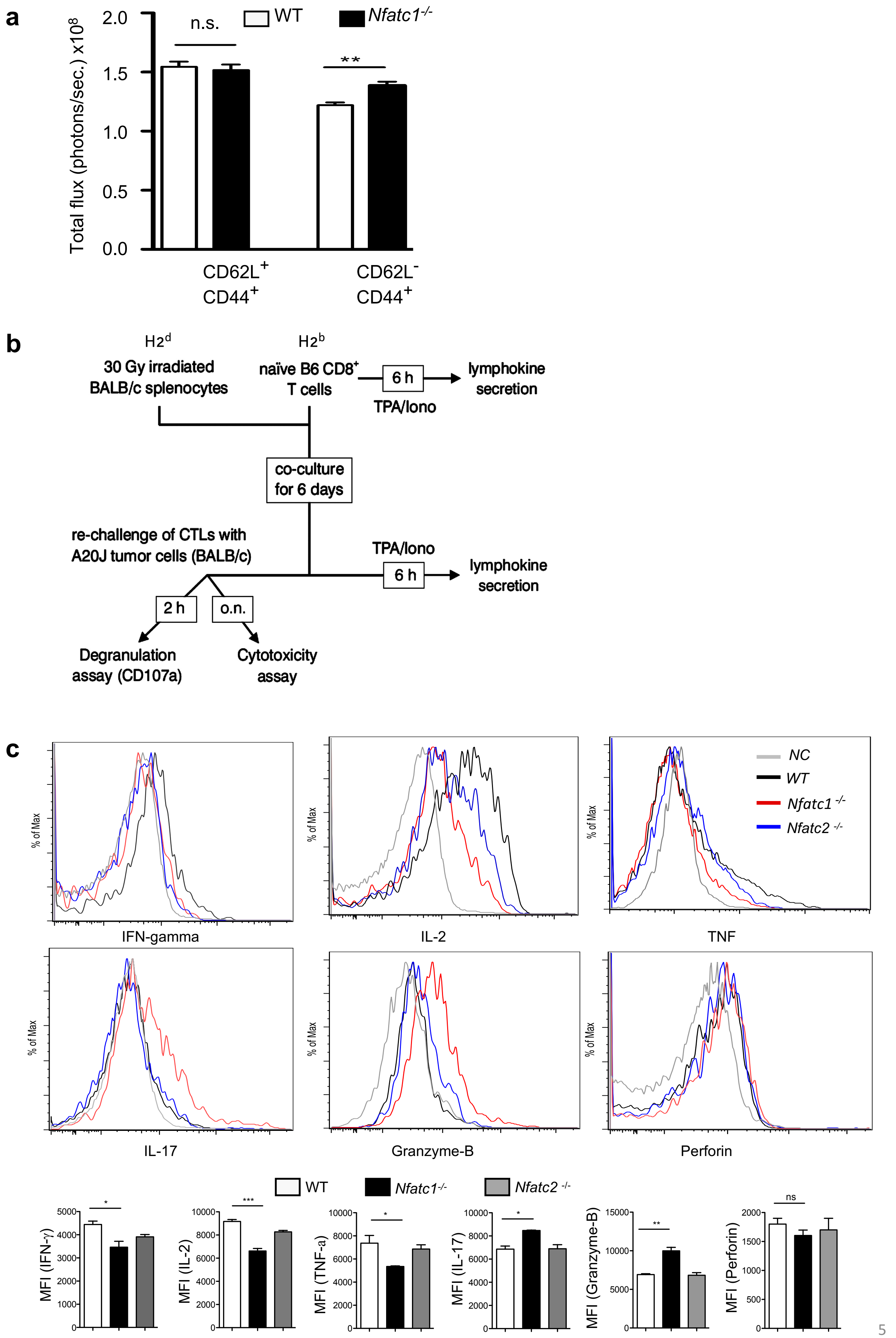
a
Total flux (photons/sec.) x10^8
2.0
1.5
1.0
0.5
0.0
n.s.
**
WT
Nfatc1-/-
CD62L+ CD44+
CD62L- CD44+
b
H2d
30 Gy irradiated BALB/c splenocytes
H2b
naïve B6 CD8+ T cells
6 h
TPA/Iono
lymphokine secretion
co-culture for 6 days
re-challenge of CTLs with A20J tumor cells (BALB/c)
TPA/Iono
6 h
lymphokine secretion
2 h
o.n.
Degranulation assay (CD107a)
Cytotoxicity assay
c
% of Max
IFN-gamma
IL-2
TNF
NC
WT
Nfatc1 -/-
Nfatc2 -/-
IL-17
Granzyme-B
Perforin
WT
Nfatc1-/-
Nfatc2 -/-
MFI (IFN-γ)
MFI (IL-2)
MFI (TNF-a)
MFI (IL-17)
MFI (Granzyme-B)
MFI (Perforin)
*
***
*
*
**
ns

d

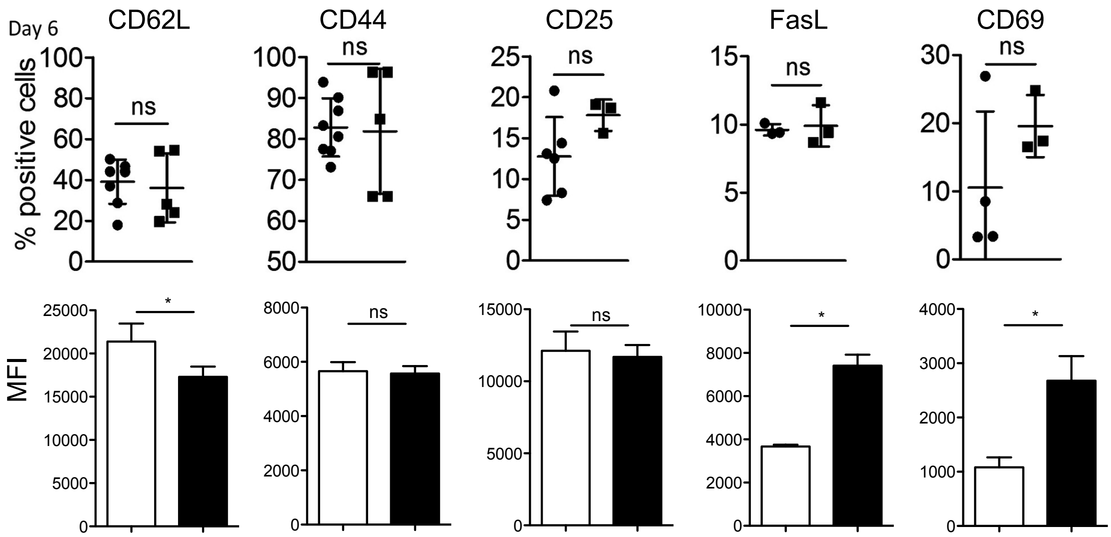

e

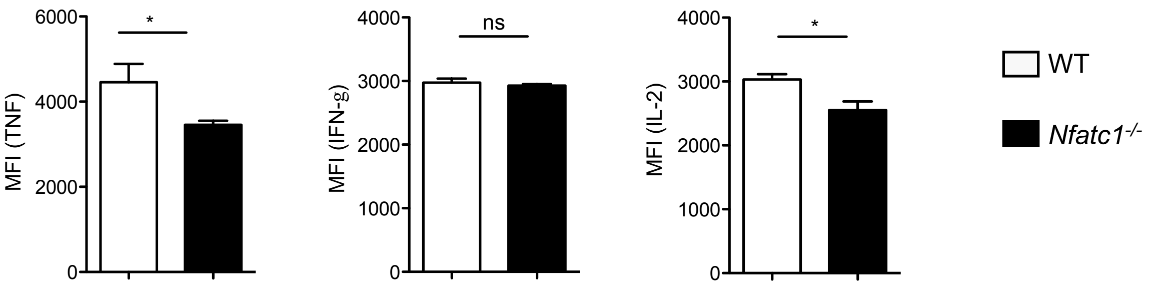

Supplementary Fig. 3 Cytotoxic activity and surface marker expression of *Nfatc1*$^{-/-}$ CTLs. **(a)** Cytotoxic activity of sorted in CD62L^{+}CD44^{+} and CD62L^{-}CD44^{+} CTLs. CTLs were sorted according to the surface markers CD62L and CD44. The sorted cells were incubated for 4 h with MOPC 315 plasmacytoma cells expressing a luciferase indicator gene. A decreased chemiluminiscence (Total flux) indicates cytotoxic activity. Data of 3 assays are shown.**(b)** Scheme of the generation of allogeneic reactive CTLs *in vitro* for cytotoxicity and degranulation assays (presented in **Fig. 2 b**). **(c)** Expression of lymphokines, granzyme B and perforin 1 in and MFI values of CTLs (see **Fig. 2c**). NC,negative control. **(d)** Expression of several surface markers and MFI values of CTLs generated for 6 d *in vitro*. ns, non-significant. **(e)** MFI values of cells shown in **Fig. 2f**. Unpaired Student's t-test was used. Data are shown as means ± SEM.

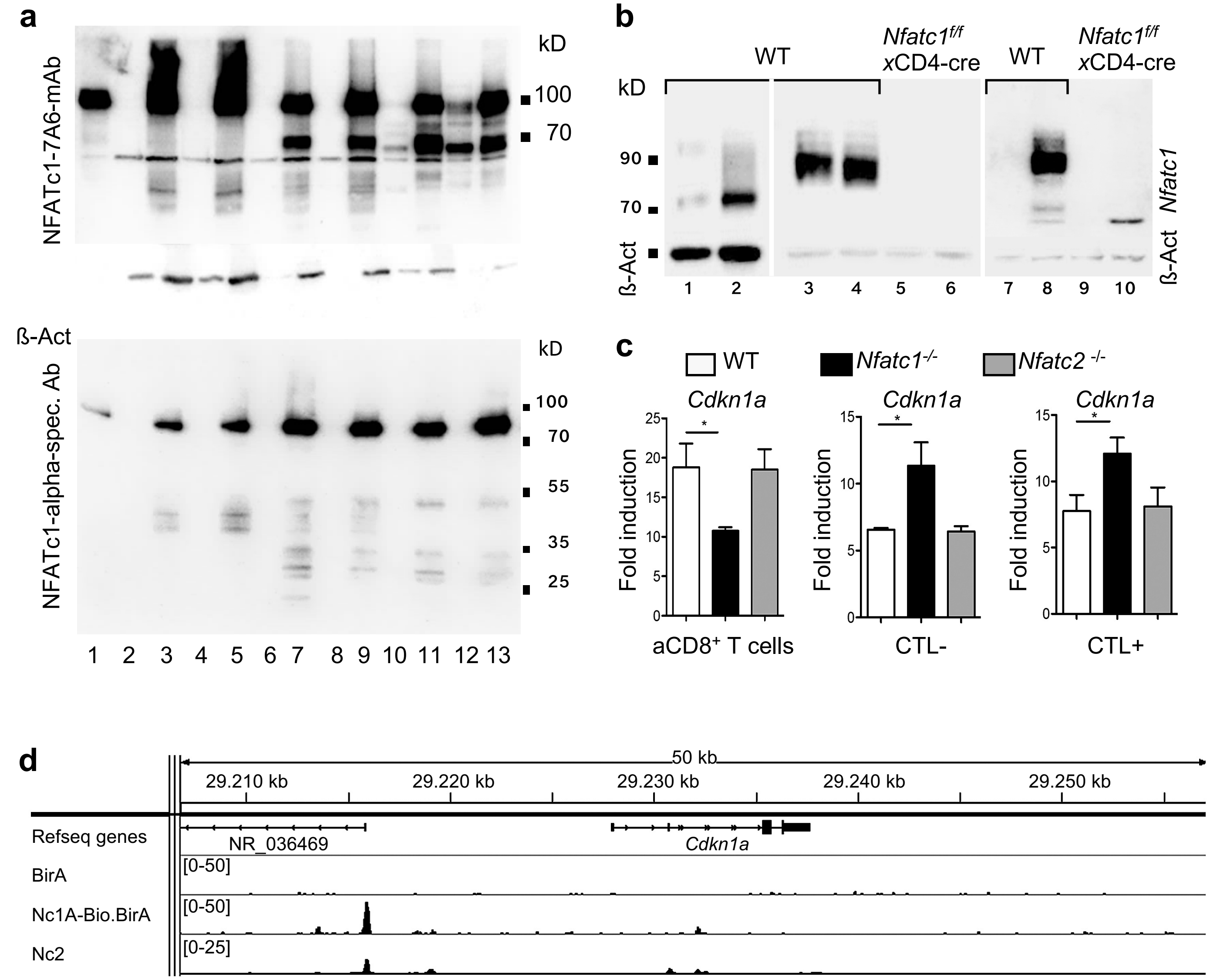

Supplementary Fig. 4 Degradation of NFATc1/αA and NFATc1-mediated control of *Cdkn1a* gene expression encoding the cell cycle inhibitor $p21^{WAW/CIP1}$. **(a)** Degradation of NFATc1/αA by protein extracts from CTLs by incubation *in vitro.* Protein from KT12 NK cells overexpressing NFATc1/αA was incubated for 30 min at 37^0 C alone (lane 1), with 30 mg nuclear protein from aCD8$^+$Ts (lane 3), from aCD8$^+$Ts treated with 100 ng/ml rapamycin (lane 5), from CTLs treated for 2 d by αCD3/CD28 and 6 d by IL-2 (lane 7), from CTLs treated for 2 d by αCD3/CD28 and 6 d by IL-2 followed by 5 h CsA (100 ng/ml) (lane 9), from CTLs treated for 2 d by αCD3/CD28 and 6 d by IL-2 and for 5 h by T+I (lane 11), or from NFATc2-deficient CTLs treated for 2 d by αCD3/CD28 and for 6 d by IL-2 and for 5 h by T+I (lane 13). In the even lanes, the protein extracts of T cells alone were fractionated. The immune blot was incubated subsequently with Ab raised against the NFATc1 α-peptide, or with the 7A6 NFATc1 Ab. One typical blot of 3 assays is shown. **(b)** Immune blots of nuclear proteins from CD8$^+$T cells of WT and *Nfatc1*$^{fl/fl\ x}$ CD4-cre mice. Cells were left unstimulated (lane 1) or stimulated with αCD3/CD28 for 6 h (lane 2), 3 d (lanes 3 and 5), or with αCD3/CD28 for 3 d followed by incubation with IL-2 for 2 d (lanes 4 and 6) or 7 d (lanes 7-10). In lanes 8 and 10, CTLs were finally stimulated by T+I for 5 h. **(c)** Real-time PCR assays of *Cdkn1a* RNA. RNAs were isolated from aCD8$^+$Ts, and from non-induced CTLs (CTL-) and CTL+ cells stimulated by T+I for 5 h. Two-tailed unpaired Student's t-test was used. Data are shown as means ± SEM. **(d)** Below, binding of NFATc1/A-Bio and NFATc2[1] to the *Cdkn1* locus. ChIP seq data are shown from CTL+ cells.

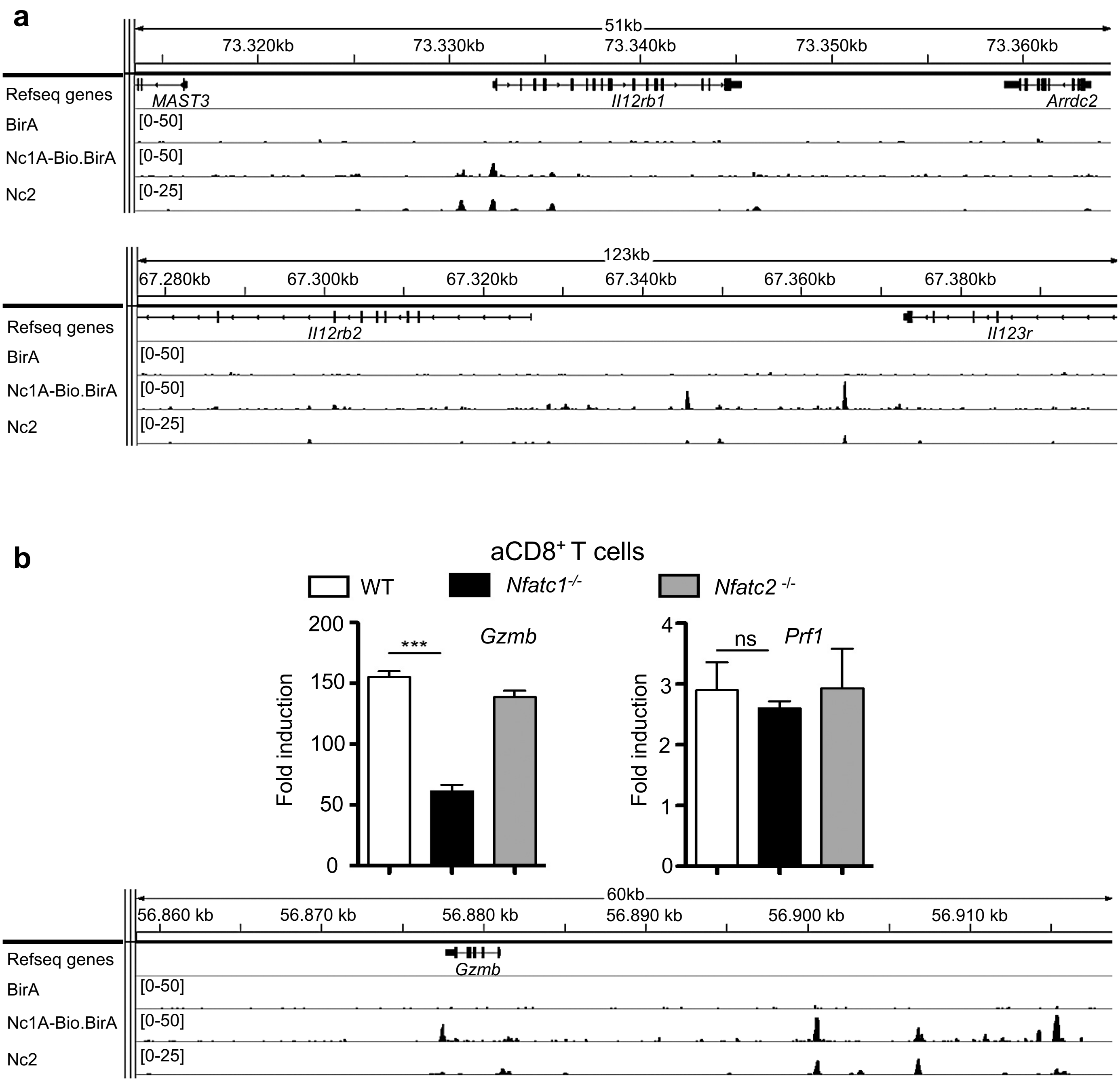

Supplementary Fig. 5 NFATc1 binding to *Il12 beta* receptor loci, and to the granzyme B locus. **(a)** Binding of NFATc1/A-Bio and NFATc2[1] to the *Il12rb* and *Il12rb2* genes encoding IL-12 receptor proteins. ChIP seq data of CTLs stimulated for 5 h by T+I. **(b)** Above, real-time PCR assays of *Gzmb* and *Prf1* RNAs encoding the effector molecules granzyme B and perforin 1, respectively. Data of 5 PCR assays are shown, relative to naïve WT CD8$^+$T cells and normalized to Actβ. Two-tailed unpaired Student's t-test was used. Data are shown as means ± SEM. RNAs were isolated from aCD8$^+$Ts. Below, binding of NFATc1/A-Bio and NFATc2[1] to the *Gzmb* gene. ChIP seq data of CTLs stimulated for 5 h by T+I.

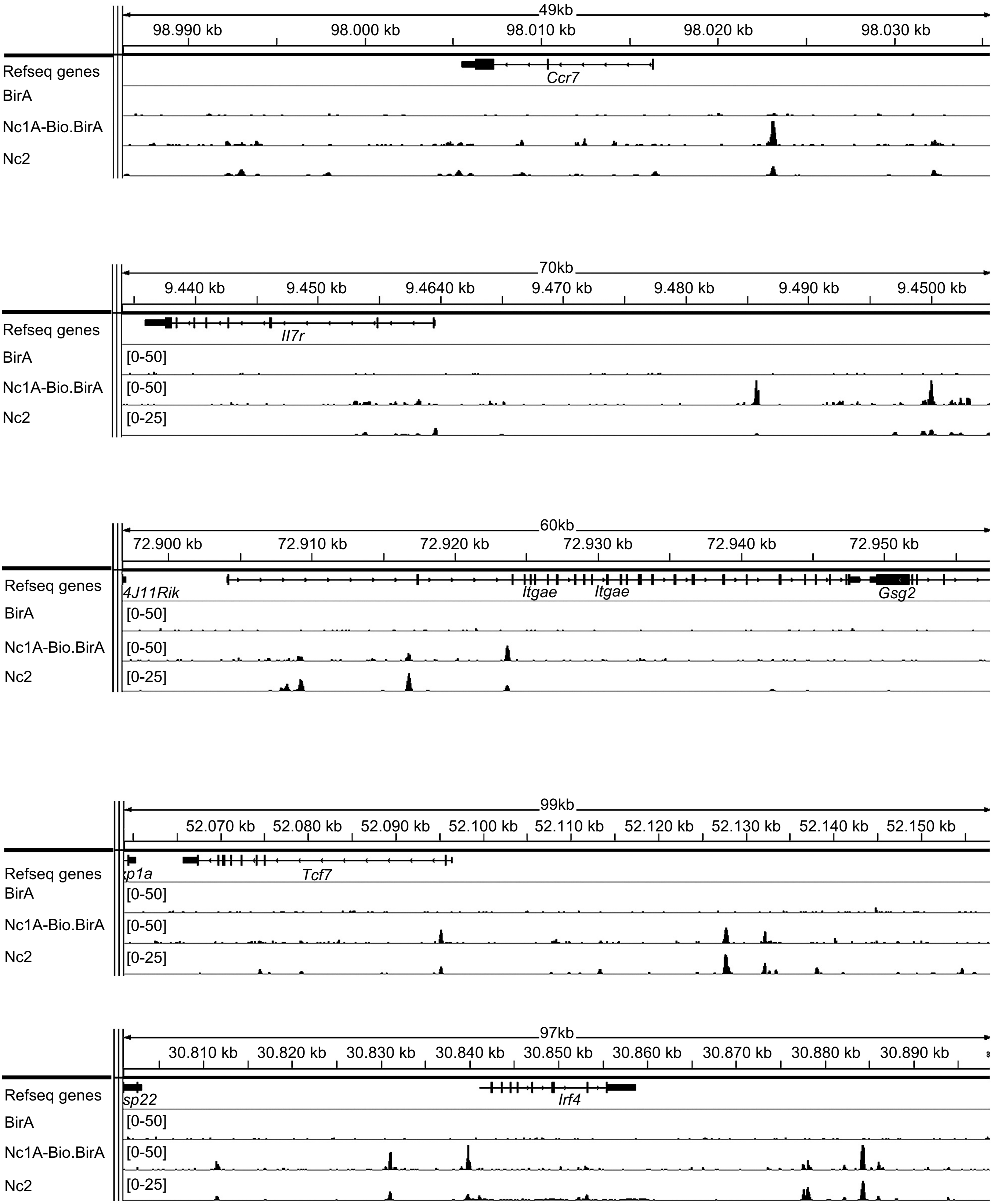

Supplementary Fig. 6 Binding of NFATc1/A-Bio and NFATc2[1] to the *Ccr7, Il7r, Itgae, Tcf7 and Irf4* loci encoding the chemokine receptor Ccr7, the IL-7 receptor α chain, the integrin CD103/integrin α_E, and the transcription factors Tcf1 and Irf4, respectively. ChIP seq data of CTLs stimulated for 5 h by T+I.

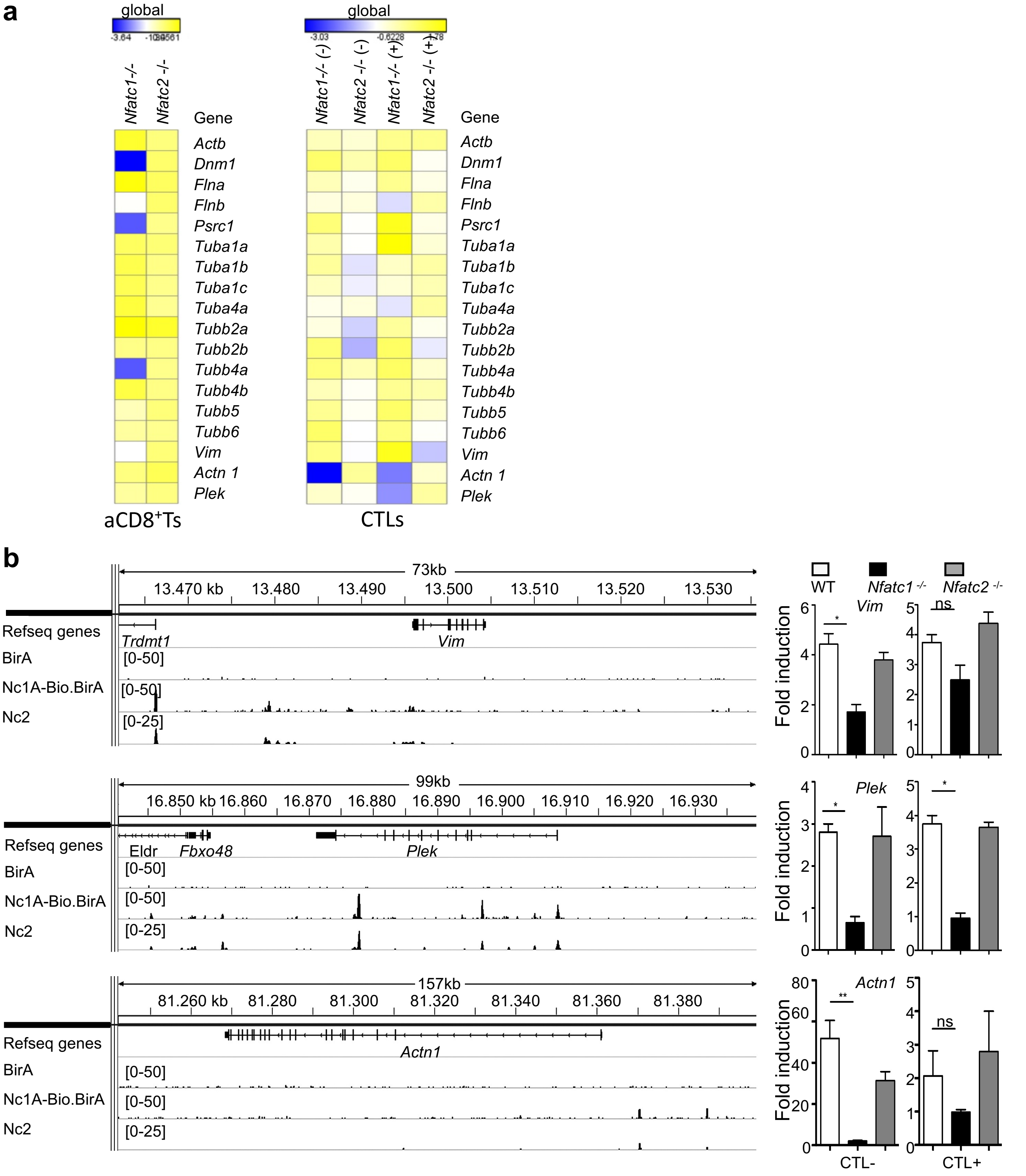

Supplementary Fig. 7 Expression of genes affecting the re-modeling of cytoskeleton in *Nfatc1*$^{-/-}$ and *Nfatc2*$^{-/-}$ CD8^{+}T cells. **(a)** Heat map of RNA expression in aCD8^{+}Ts (left) and CTLs (right) of selected genes encoding cytoskeleton proteins. NGS transcriptome data of *Nfatc1*$^{-/-}$ and *Nfatc2*$^{-/-}$ aCD8^{+}Ts and CTLs are shown, relative to WT cells. **(b)** Left, binding of NFATc1/A-Bio and NFATc2[1] to the *Vim, Plek* and *Actn1* loci in CTL+ cells. ChIP seq assays. Right, real-time PCR assays of *Vim, Plek* and *Actn1* RNAs encoding vimentin, pleckstrin and α-actinin 1, respectively. RNAs were isolated from CTLs. Data of 5 PCR assays are shown, relative to naïve WT CD8^{+}T cells and normalized to Actβ. Two-tailed unpaired Student's t-test was used. Data are shown as means ± SEM.

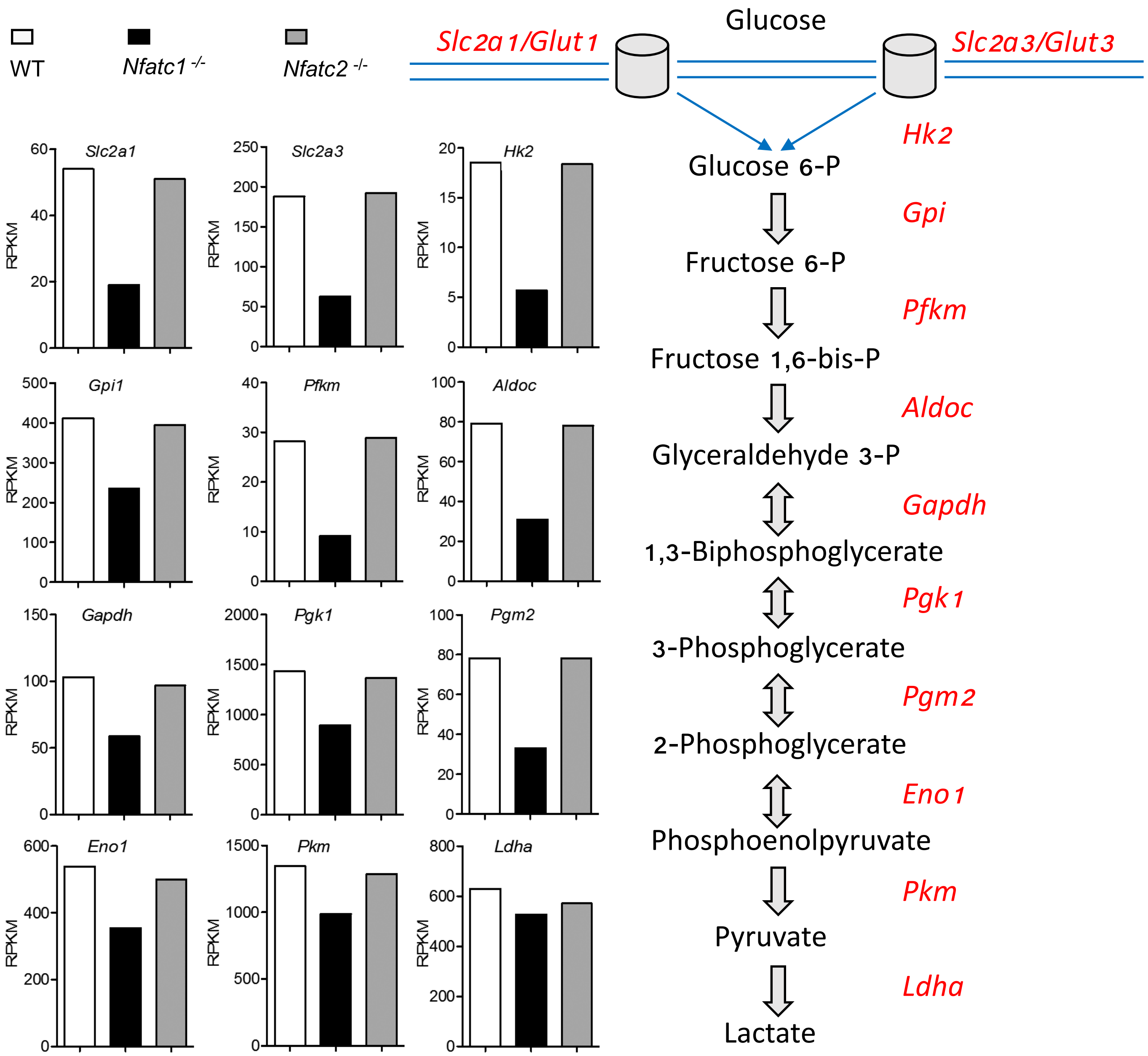

Supplementary Fig. 8 Effect of NFATc1 and NFATc2 ablation on the RNA expression of genes of aerobic glycolysis in aCD8^{+}T cells. NGS results of transcriptome assays.

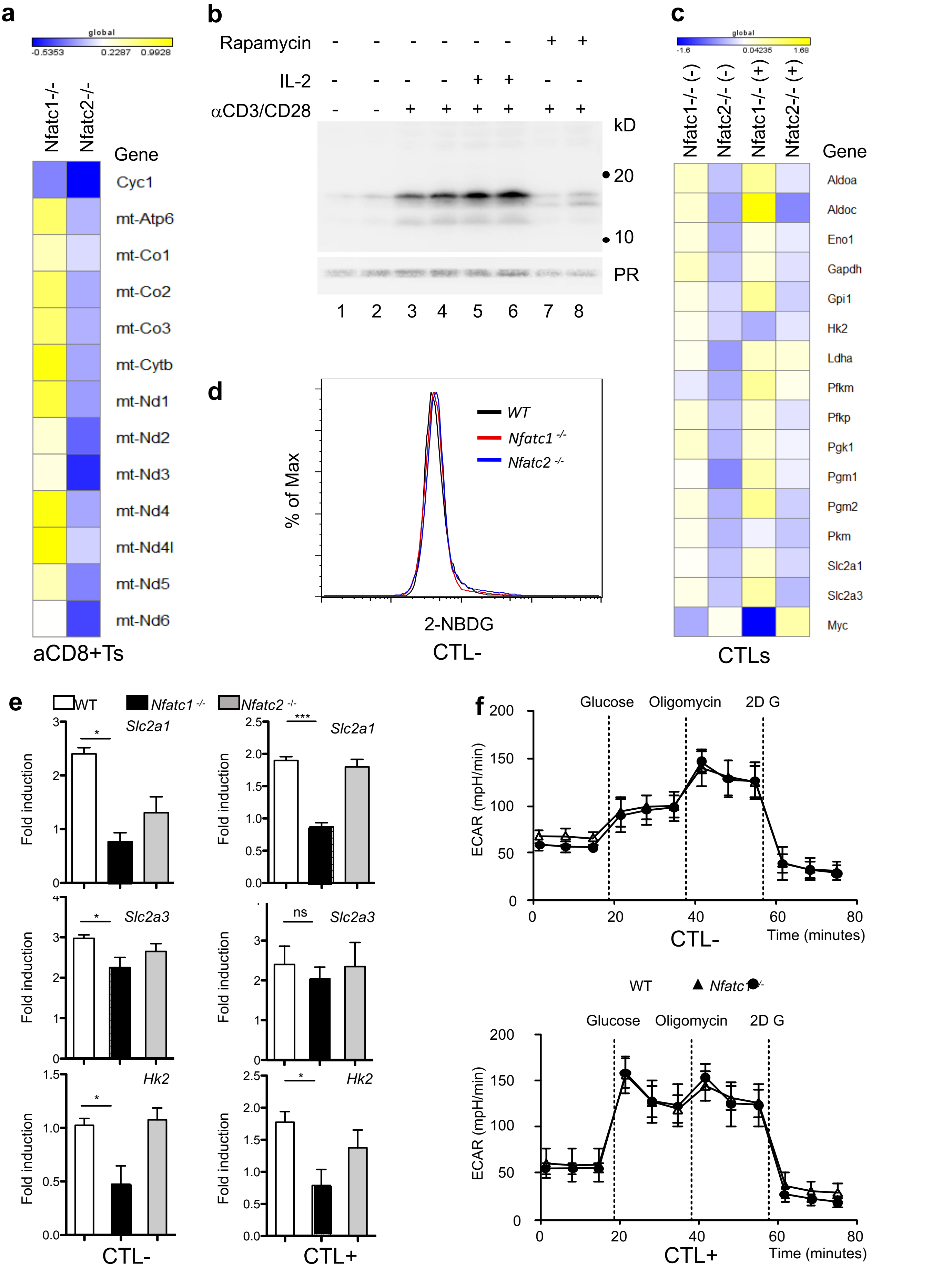

Supplementary Fig. 9 Effect of NFATc1 ablation on metabolism of CTLs. **(a)** Heat map of RNA expression of mitochondrial genes in aCD8⁺T cells, compared to WT cells. For comparison, the expression of nuclear cytochrome c-1 gene (*Cyc1*) is also shown. **(b)** Immune blot showing the

effect of αCD3/CD28, IL-2 and Rapamycin on the Thr37/46 phosphorylation of the mTOR target 4E-BP1 in splenic WT and *Nfatc1*$^{-/-}$ CD8^{+}T cells upon treatment for 24 h. **(c)** Heat map of RNA expression of genes of glycolysis cascade in *Nfatc1*$^{-/-}$ and *Nfatc2*$^{-/-}$ CTL- and CTL+ cells. **(d)** Incorporation of 2-NBDG into CTL- cells upon incubation for 1 h at 37°C. One typical assay of 2 experiments is shown. **(e)** Real-time PCR assays of *Slc2a1, Slc2a3* and *Hk2* RNAs encoding the glucose transporters Glut1 and Glut3, and hexokinase 2, respectively, isolated from CTL- and CTL+ cells. Data of 5 PCR assays are shown, relative to naïve WT CD8^{+}T cells and normalized to Actβ. **(f)** Extracellular flux analysis. 4x10^{5} CTL- or CTL+ cells were seeded in a plate pre-coated with poly-D-lysine (Sigma; 50 mg/ml) and subjected to extracellular flux analysis. Typical assays of 3 experiments are shown. For (e), two-tailed unpaired Student’s t-test was used. Data are shown as means ± SEM.

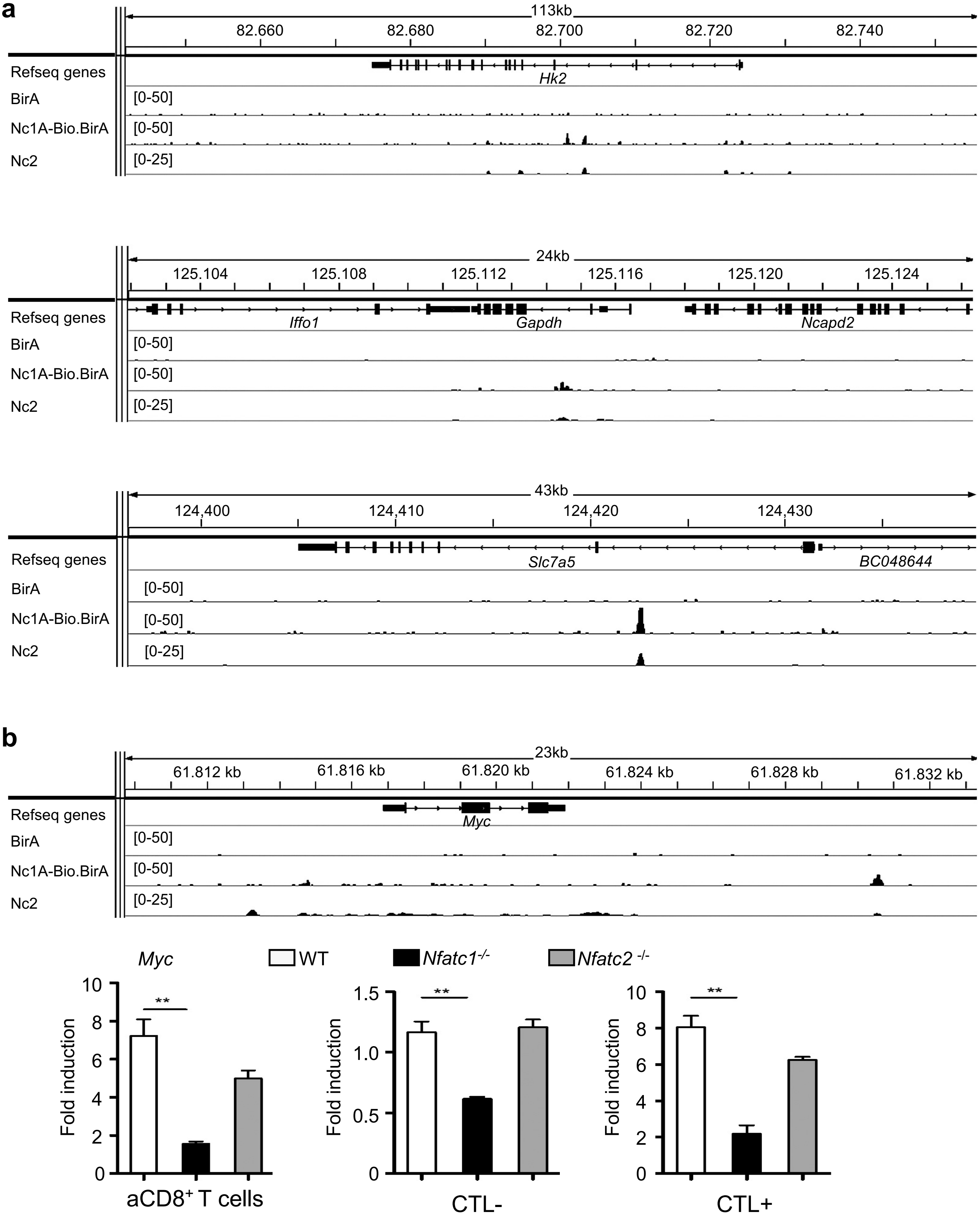

Supplementary Fig. 10 NFATc1 binding to the *Hk2, Gapdh, Slc7a5 and Myc* loci encoding hekokinase 2, glyceraldehyde 3-phosphate dehydrogenase, cationic aa transporter Slc7a5 and c-Myc, respectively. **(a)** ChIP seq assays of NFATc1/A-Bio and NFATc2[1] binding to the *Hk2, Gapdh* and *Slc7a5* genes in CTL+ cells. **(b)** Above, ChIP seq assays of NFATc1/A-Bio and NFATc2[1] binding to the *Myc* locus in CTL+ cells. Below, real time PCR assays of *Myc* RNA. Data of 5 PCR assays are shown, relative to naïve WT CD8$^+$T cells and normalized to Actβ.Two-tailed unpaired Student's t-test was used. Data are shown as means ± SEM.

Reference

1) Martinez, G. J. *et al.* The transcription factor NFAT promotes exhaustion of activated CD8(+) T cells. *Immunity* **42**, 265-278, doi:10.1016/j.immuni.2015.01.006 S1074-7613(15)00032-1 [pii] (2015).

Supplementary Table 1

List of Real Time-PCR Primers

Gene	Primer Sequence
Ifng	For: 5'-GAGCTCATTGAATGCTTGGC Rev: 5'-GCGTCATTGAATCACACCTG
Cxcr3	For: 5'-TCTCGTTTTCCCCATAATCG Rev: 5'-AGCCAAGCCATGTACCTTGA
Ccr7	For: 5'-GTCTCTCTCCAGCTAGCCCA Rev: 5'-CAAACAGGAGCTGATGTCCA
Cdkn1a	For: 5'-ACGGGACCGAAGAGACAAC Rev: 5'-CAGATCCACAGCGATATCCA
Slc2a3	For: 5'-: ATCGTGGCATAGATCGGTTC Rev: 5'-CCGCTTCTCATCTCCATTGT
Cdkn2a	For: 5'-GCAGAAGAGCTGCTACGTGA Rev: 5'-CGTGAACATGTTGTTGAGGC
Cad	For: 5'-TACGCAGTTCTCATCGACCA Rev: 5'-TGGGAGTTGCATGAAGAGTG
Myc	For: 5'-ACGGAGTCGTAGTCGAGGTC Rev: 5'-AGAGCTCCTCGAGCTGTTTG
Sell	For: 5'-TTCATGGCTTTCCTTTCACA Rev: 5'-CTGGCATTTCTCATTTGGCT
Gls2	For: 5'-AGTTCACCACGGCTCTGAAG Rev: 5'- CACACCTGGATCCCAGACAC
Slc1a5	For: 5'-GGACGTCTTTCATCTCCACAA Rev: 5'-ACTCCTTCAATGATGCCACC
Cdkn1b	For: 5'-GGGGAACCGTCTGAAACATT Rev: 5'AGTGTCCAGGGATGAGGAAG
Il7r	For: 5'CATTTCACTCGTAAAAGAGCCC Rev: 5'-TGGAAGTGGATGGAAGTCAA
Ccl3	For: 5'-GTGGAATCTTCCGGCTGTAG Rev: 5'-ACCATGACACTCTGCAACCA
Ccl4	For: 5'-GAAACAGCAGGAAGTGGGAG Rev: 5'-CATGAAGCTCTGCGTGTCTG
Il2	For: 5'-CGCAGAGGTCCAAGTTCATC Rev: 5'-AACTCCCCAGGATGCTCAC
Itgae	For: 5'-GCCCAGTCCACATCCATATT Rev: 5'-GCTGCATCTGCTCCAGCTAT
Slc2a1	For: 5'-GAGTGTGGTGGATGGGATG Rev: 5'-AACACTGGTGTCATCAACGC
Ccr7	For: 5'-ACACAGGAAGGCTGTGCTTT Rev: 5'-CATGGACTGCTATCTGCGTC
Actn1	For: 5'-TCGGAAGTCCTCTTCGATGT Rev: 5'GGGAGAAGCAGCAGAGGAAG
Gzmb	For: 5'- CATGTAGGGTCGAGAGTGGG Rev: 5' CCTCCTGCTACTGCTGACCT
Prf1	For: 5'- TGGAGGTTTTTGTACCAGGC Rev: 5' TAGCCAATTTTGCAGCTGAG
Eomes	For: 5'- GACCTCCAGGGACAATCTGA Rev: 5' GGCCTACCAAAACACGGATA
Hk2	For: 5'- GGAACCGCCTAGAAATCTCC Rev: 5'- GGAGCTCAACCAAAACCAAG
Tbx21	For: 5'- ATCCTGTAATGGCTTGTGGG Rev: 5'-TCAACCAGCACCAGACAGAG
Irf4	For: 5'- CAAAGCACAGAGTCACCTGG Rev: 5' TGCAAGCTCTTTGACACACA
Plek	For: 5'- CCACATGGGTTTCCAGGTAT Rev: 5'- AAGAGTGGACCCGTGTGTCT
Vim	For: 5'- TCCACTTTCCGTTCAAGGTC Rev: 5'- AGAGAGAGGAAGCCGAAAGC
